# Electrospun Fibrous Silica for Bone Tissue Engineering Applications

**DOI:** 10.3390/pharmaceutics15061728

**Published:** 2023-06-14

**Authors:** Alexandra Elena Stoica (Oprea), Alexandra Cătălina Bîrcă, Oana Gherasim, Anton Ficai, Alexandru Mihai Grumezescu, Ovidiu-Cristian Oprea, Bogdan Ștefan Vasile, Cornel Balta, Ecaterina Andronescu, Anca Oana Hermenean

**Affiliations:** 1 Department of Science and Engineering of Oxide Materials and Nanomaterials, University Politehnica of Bucharest, 011061 Bucharest, Romaniaada_birca@yahoo.com (A.C.B.); anton_ficai81@yahoo.com (A.F.); grumezescu@yahoo.com (A.M.G.); 2 Lasers Department, National Institute for Lasers, Plasma and Radiation Physics, 409 Atomistilor Street, 077125 Magurele, Romania; 3 Research Institute of the University of Bucharest—ICUB, University of Bucharest, 050657 Bucharest, Romania; 4 Academy of Romanian Scientists, Ilfov No. 3, 050044 Bucharest, Romania; ovidiu73@yahoo.com; 5 Department of Inorganic Chemistry, Physical Chemistry and Electrochemistry, Faculty of Applied Chemistry and Materials Science, University Politehnica of Bucharest, 1-7 Gheorghe Polizu Street, 011061 Bucharest, Romania; 6 National Research Center for Micro and Nanomaterials, University Politehnica of Bucharest, 060042 Bucharest, Romania; bogdan.vasile@upb.ro; 7 HTP Research and Consulting, Joita, 087150 Giurgiu, Romania; 8 Research Center for Advanced Materials, Products and Processes, University of Bucharest, 060042 Bucharest, Romania; 9 “Aurel Ardelean” Institute of Life Sciences, Vasile Goldiş Western University of Arad, 310025 Arad, Romania; baltacornel@gmail.com (C.B.); ancahermenean@gmail.com (A.O.H.)

**Keywords:** fibrous silica, electrospinning, tissue regeneration

## Abstract

The production of highly porous and three-dimensional (3D) scaffolds with biomimicking abilities has gained extensive attention in recent years for tissue engineering (TE) applications. Considering the attractive and versatile biomedical functionality of silica (SiO_2_) nanomaterials, we propose herein the development and validation of SiO_2_-based 3D scaffolds for TE. This is the first report on the development of fibrous silica architectures, using tetraethyl orthosilicate (TEOS) and polyvinyl alcohol (PVA) during the self-assembly electrospinning (ES) processing (a layer of flat fibers must first be created in self-assembly electrospinning before fiber stacks can develop on the fiber mat). The compositional and microstructural characteristics of obtained fibrous materials were evaluated by complementary techniques, in both the pre-ES aging period and post-ES calcination. Then, in vivo evaluation confirmed their possible use as bioactive scaffolds in bone TE.

## 1. Introduction

Aiming to overcome the bioavailability-related limitations and immunogenicity-related complications of conventional transplantation, tissue engineering (TE) enables the elaboration and fabrication of functional materials and technologies that mimic the structural configuration of natural tissues and exert an increased ability to successfully restore or replace biological functions [1,2]. At present, the biggest challenge for the reconstruction and regeneration of human tissues can be conveniently met using advanced materials science, biomolecular and cellular engineering, stem cell science, and nanotechnology, which define the multidisciplinary archetype to design complex and personalized therapeutic strategies [3,4].

To properly modulate the reparative or regenerative events of tissues, it is essential for the three-dimensional (3D) artificial constructs (scaffolds) to simulate the intrinsic properties of natural extracellular matrices (ECMs). A successful strategy to meticulously mimic their hierarchical structure and features is to design and fabricate biomaterials with a nanoscale topographical appearance, micro- and macroscale gradient structure, and intricate and interconnected morphology, which provide proper cellular migration and normal cellular development, oxygenation, nutrition and vascularization [5,6,7]. More than simply providing the appropriate compositional, structural, and biomechanical support for specific tissue formation, the biofunctional performance of tissue-engineered artificial architectures can be enhanced by seeding cells or immobilizing growth factors into the material’s microstructure [8,9].

Regarding the reparative or regenerative potential of nanotechnology-derived platforms, impressive outcomes have been reported for different types of one-dimensional nanomaterials, such as nanofibers [10,11], nanowires [12,13], and nanotubes [14,15]. Nanofibers have gained a lot of interest within academia and industry due to their high surface area-to-volume ratio, high aspect ratio, flexibility and ease of tailoring their surface properties and functionalities, and superior mechanical properties. Furthermore, nanofibers as building blocks for nanomembranes and nanodevices find prospects for applications in various modern domains, including biomedical research, drug delivery, textiles, filtration, catalysis, energy storage, sensors, and electronics [4,5,16,17].

Different methods have been successfully employed for the production of nanofibers, including phase separation [18], melt blowing [19], drawing [20], template synthesis [21], self-assembly [22], and electrospinning (ES) [23,24]. Among these, ES is an adaptable and versatile technique for fabricating nanofibers from a large variety of materials (ceramics, polymers, and metals), in a rapid and controlled mode [16,25].

ES generates ultrathin fibers with diameters ranging from tens of nanometers to few micrometers, and the potential clinical use of this technology in tissue repair and regeneration has been thoroughly confirmed in recent years [26,27,28,29,30,31]. It is expected that research into ES-fabricated platforms will become highly interdisciplinary in the near future, especially with regard to their impressive results in tissue engineering and regenerative medicine. A deeper understanding of the bidirectional interactions between ES-generated constructs and as-treated hosts is necessary to demonstrate the effects of nanofibers on the biochemical pathways and cellular signaling mechanisms that control cell morphology, differentiation, growth, proliferation, motility, and genotype, as well as understanding how the natural ECM components dictate the fate of synthetic 3D scaffolds [28,32].

Despite the considerable number of scaffolding techniques, electrospinning provides an attractive and remarkable alternative to producing non-woven fibrous structures (coatings and membranes, scaffolds and sponges, complex constructs) [33,34,35] with dimensional, morphological and biofunctional characteristics comparable to those of natural ECM environments. As nanofibers with specific and selective functionality can be obtained by ES (thus conforming to the particular demands of the tissue to be restored or replaced), impressive outcomes have recently been reported for ES-generated platforms in TE applications [26,36,37,38].

Inorganic ceramics possess excellent thermal resistance, high rigidity, and wear resistance, and superior oxidative and chemical resistance, all of which make them appropriate choices for use under critical conditions [39]. Silica (SiO_2_) is an available and low-cost material that has been largely used. Since the introduction of silica-based materials in the early 1990s by scientists of the Mobil Corporation and Waseda University, these materials have gained tremendous interest in research and industrial applications. Owing to their large surface area and pore volume ratio, uniform and well-structured porosity, size-related reactivity, and tunable surface properties, such nanostructured mesoporous systems have been broadly used in catalysis (as host matrices for different guest molecules), environmental remediation (as photocatalysts and decontaminating agents), agri-food industry (as absorbers, strengtheners, and preservatives), and biomedicine (as bioactivity modulators, bioimaging enhancers, and pharmacotherapeutic formulations) [40,41,42,43,44].

Recently, the promising biomedical utilization of mesoporous silicas as active molecule carriers has been extensively investigated for bone tissue repair and regeneration purposes [45]. Silica (SiO_2_) is an abundantly available and low-cost material that has been largely explored for bone tissue engineering and regenerative medicine. Besides being a major source of silicon (which exerts regulatory effects during bone homeostasis, neocollagenesis, and matrix mineralization) [44,46,47,48], SiO_2_ nanostructures are highly efficient loading/releasing transporters for therapeutic biomolecules [49,50] and active targeting molecules [51,52].

When obtained into fibers, bulk fibrous silica presents robust properties with promising use in catalysis, adsorption, encapsulation, sensors, and integrated circuits. Though SiO_2_ nanostructured constructs are able to induce and potentiate bone repair and regeneration [39,53], obtaining silica fibers with diameters in the submicron and nanometer scale, as well as three-dimensional structures, is still challenging [4,54,55,56].

In this study, we found that electrospun fibers could self-assemble into 3D stacks using specific condensed TEOS/PVA (tetraethyl orthosilicate/polyvinyl alcohol) solutions under mild electrospinning conditions (namely, room temperature and humidity). Subsequently, 3D uniform fibrous networks based on pure silica could be easily obtained by calcination then used as bone scaffolds (as evidenced by their regeneration ability on murine calvarial defects.

## 2. Materials

Reagent-graded tetraethyl orthosilicate (TEOS), poly(vinyl alcohol) (PVA, Mw = 89,000–98,000), and phosphoric acid (H_3_PO_4_) solution (85 wt% in H_2_O) were purchased from Sigma-Aldrich (Merck Group, Darmstadt, Germany). All chemicals were of analytical purity and used in our experiments with no further purification. Deionized water (DIW) was used throughout the experiments.

### 2.1. Preparation of TEOS/PVA Solution

Each silica precursor solution was prepared by mixing TEOS and DIW (1.1:1 volume ratio), then adding drop-by-drop a reduced volume of H_3_PO_4_ solution, followed by room temperature stirring for 3.5 h to obtain a homogenous hydrolyzed solution. Polymer solutions were prepared by dissolving PVA in DIW (150 mg/mL concentration) at 50 °C for 1 h. The as-obtained solutions were mixed and stirred for 2 h at room temperature, and then the TEOS/PVA mixtures were heated at 60 °C for 1.5 h, aged for different periods (0 h and 1.5 h), and further used for electrospinning.

### 2.2. Electrospinning Deposition of Silica-Based Materials

The previously prepared solutions were loaded into a polypropylene syringe connected to an 18-gauge blunt-end needle and then mounted on a digital syringe pump. The electrospinning procedure was carried out using Tong Li Tech (Shenzhen, China) ES equipment at 24 kV voltage (−4 kV and 20 kV), 200 mm needle-to-target distance, and 10 mL/h flow rate for 30 min for all solutions. The TEOS/PVA fibers were directly deposited on a piece of grounded aluminum foil (pristine electrospun SiO_2_/PVA_0h and SiO_2_/PVA_1.5h aging mats). To obtain silica (SiO_2_) fibers, the as-spun TEOS/PVA networks were heated up to 500 °C (with a 10 °C/min heating rate) and air-dried for 2 h in a Tong Li Tech furnace (calcined electrospun SiO_2__0h and SiO_2__1.5h aging mats).

### 2.3. Physico-Chemical Characterization of Electrospun Networks

#### 2.3.1. Fourier Transform Infrared Spectroscopy (FT-IR)

FT-IR spectra were obtained with a FT-IR spectrometer Nicolet 6700 from Thermo Nicolet (Wisconsin, MA, USA). The samples were analyzed by a ZnSe crystal, and measurements were executed through 32 sample scans between 400 and 4000 cm^−1^ and at a resolution of 4 cm^−1^ at room temperature. In order to be able to register the acquired information, the spectrometer was connected to a data acquisition and processing unit through the Omnic program (version 8.2 Thermo Nicolet).

#### 2.3.2. Thermal Analysis (TGA and DSC)

The thermal analysis was performed with a STA 449C Jupiter equipment from Netzsch (Selb, Germany). To perform simultaneous thermogravimetric analysis (TGA) and differential scanning calorimetry (DSC), samples were placed in open alumina crucibles and heated with 10 K/min from room temperature to 900 °C, under 50 mL/min air flow.

#### 2.3.3. Scanning Electron Microscopy (SEM)

To investigate the microstructure of synthesized samples, scanning electron micrographs were collected using the secondary electron beam (30 keV energy) of a Quanta Inspect F50 FEI, equipped with energy-dispersive X-ray spectroscopy (EDS) accessory, a microscope purchased from Thermo Fischer Scientific (OR, Waltham, MA, USA). All samples were capped with a thin gold layer before investigation.

#### 2.3.4. Transmission Electron Microscopy (TEM)

To obtain relevant information on the intimate microstructure of electrospun samples, transmission electron micrographs were acquired using a high-resolution Tecnai G2 F30 S-TWIN microscope purchased from Thermo Fisher Scientific (former FEI, Hillsboro, OR, USA). In this respect, small amounts from all specimens were placed onto a shell-like copper grid and investigated in the transmission mode at an accelerating voltage of 300 kV.

### 2.4. Biological Evaluation of Fibrous Networks in Bone Tissue Engineering Applications

#### 2.4.1. Animal Model and Surgical Procedure

**Figure 1:**
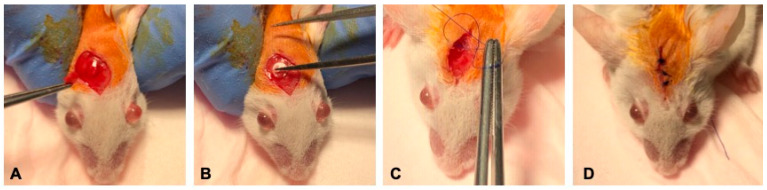
Surgical procedure. (**A**) Execution of the 5 mm critical size defect in the rat calvarium. (**B**) Placement of the silica-based fibrous scaffolds. (**C**,**D**) Closure of the periosteum and overlaying skin.

Adult CD1 mice were used throughout the experiments (Figure 1), which were approved by the Ethics Committee for Research of the Vasile Goldis Western University of Arad. The animals were placed under anesthesia during the critical bone defect surgery following the intraperitoneal (i.p.) administration of ketamine/xylazine mixture (100 mg/kg and 10 mg/kg body weight, respectively). After anesthesia, 5 mm full-thickness calvarial defects were prepared using a 3.5 mm power drill under constant saline solution irrigation, as previously described [57]. The periosteum reflected over the defect site, and the incision was closed. No lethality was detected during the surgery or over the post-surgical period. After surgery, the animals were housed individually under constant conditions.

To respect the European regulations regarding animal experiments (3R rules), only 2 of the 4 prepared samples were used, namely the non-calcined and calcined aged specimens. The silica-based fibrous mats were cut into disks using a 5 mm biopunch, then sterilized by ultraviolet (UV) exposure for 20 min. The mice were randomly divided into distinctive groups, as Group 1 (SiO_2_/PVA_1.5h aging), Group 2 (SiO_2_/PVA_1.5h aging + VN), Group 3 (SiO_2__1.5h aging), and Group 4 (SiO_2__1.5h aging + VN). To achieve an uniform distribution of the vitronectin (VN) within the fibrous networks, samples were placed in sterile 24 well-plates, inoculated with VN solution (5 µg of recombinant murine vitronectin [58] added to 50 µL of sterile water) and left at 40 °C for 24 h.

The animals were euthanatized after 4 weeks (*n* = 10 animals), using an overdose of xylazine-ketamine, and the implants were harvested for subsequent evaluation.

#### 2.4.2. High-Resolution X-ray

Radiographs from the implantation sites were taken using the XTREME in vivo imaging system from Carestream Health (Rochester, NY, USA).

#### 2.4.3. Histopathology

The calvarial new tissue with the surrounding bone and soft tissue was washed with phosphate-buffered saline (PBS) and fixed in paraformaldehyde (PFA) solution (4% in PBS) for 3 days. Decalcification was performed in Biodec R (Bio-Optica, Milan, Italy) for 5 days, at room temperature. Next, the samples were dehydrated, cleared, and embedded in paraffin blocks.

Histological sections (5 μm) were prepared using a microtome then subsequently stained with Gomori’s trichrome (Leica Biosystems, Wetzlar, Germany) and Alizarin Red (anthraquinone derivative used to identify calcium deposits), to evaluate tissue histology and mineralization. More, Hematoxylin and Eosin (H&E, Bio-Optica) staining was performed as the routine histological protocol used for pathological and morphological examination. Hematoxylin acts as a very strong basic dye used to stain the nuclei in blue, while eosin is an acid dye that stains the cytoplasm in red or pink.

All slides were examined by light microscopy using an Olympus BX43 microscope equipped with an Olympus XC30 digital camera purchased from Olympus Life Science (Tokyo, Japan).

## 3. Results and Discussions

The electrospinning (ES) technique remains the most advantageous method to obtain nano-/micro-fibers in various patterns, representing a versatile and focused-upon strategy to fabricate modern solutions for current day-to-day challenges, and has gathered more than 32,000 publications to date (Scopus). In particular, the attractive and tunable features of ES-fabricated systems in the framework of modern bionanotechnology have considerably boosted the progress of personalized biomedicine by providing an efficient and easy method to produce nanomaterials, nanoplatforms and nanodevices for tissue engineering and regenerative medicine [59,60].

The impressive biomedical potential of silica-based nanomaterials relies on their excellent bioactivity, which is strongly connected to their composition and microstructure, and enables the development of performance-enhanced therapeutic formulations [61,62]. The morphology and size, porosity, and surface area of silica (SiO_2_) networks can be easily optimized during the synthesis protocol. Although the use of silica as filler in different composite scaffold matrices has been successfully validated for tissue engineering applications [63,64,65], there are few papers regarding the fabrication and biofunctional evaluation of 3D scaffolds based exclusively on silica.

### 3.1. Physico-Chemical Investigation of Electrospun Networks

**Figure 2:**
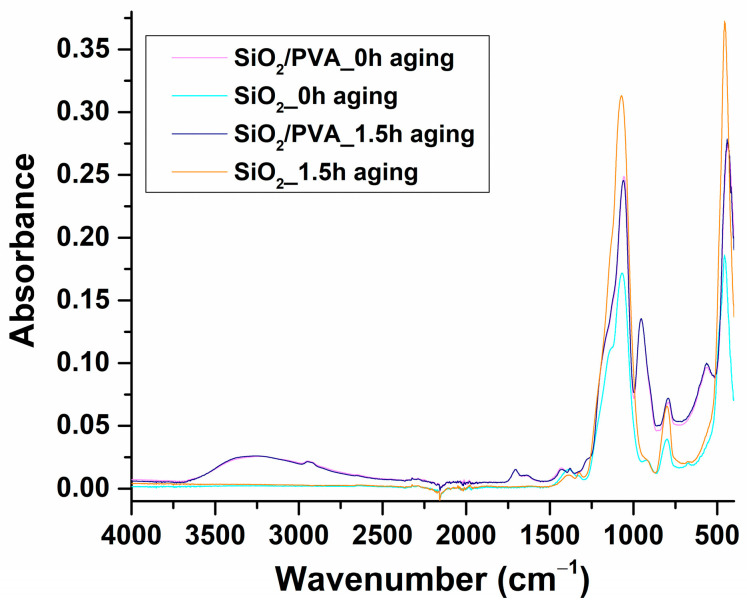
FT-IR spectra recorded for SiO_2_/PVA_0h aging (pink), SiO_2__0h aging (blue), SiO_2_/PVA_1.5h aging (purple), and SiO_2__1.5h aging (orange).

To investigate the composition of ES-obtained samples (by identifying the characteristic absorption bands and the interactions between compounds), FT-IR spectra were recorded for non-aged and 1.5 h aged materials (Figure 2), both before (SiO_2_/PVA_0h and SiO_2_/PVA_1.5h aging) and after 500 °C treatment (SiO_2__0h aging and SiO_2__1.5h aging).

The infrared spectrum of non-aged and non-calcined samples (Figure 2, pink) shows the preponderant presence of silica-originating vibrations, such as asymmetric Si–O–Si stretching (~1060 cm^−1^), overlapped Si–O–C stretching and symmetric Si–O–Si stretching (~800 cm^−1^), Si–OH bending (~950 cm^−1^), and Si–O stretching (~440 cm^−1^) [66,67]. Besides Si-containing bonds, the intense absorbance maxima from ~1060 cm^−1^ may superpose the vibrations of C–C–O from the ethoxy of TEOS and ethanol (a hydrolysis product), while the subtle shoulder between 1100–1200 cm^−1^ may be assigned to the C–O stretching from PVA [68,69]. Additionally, the presence of carbon-containing bonds originating from PVA is noticed at ~2900 cm^−1^ and ~1700 cm^−1^ [69,70]. The hydroxyl group contribution is observed as a wide absorption band in the 3000–3400 cm^−1^ wavenumber range.

Following calcination at 500 °C, the IR data (Figure 2, blue) indicate the absence of all peaks corresponding to PVA, proving that organic molecules can be completely removed from silica/PVA composites starting from this temperature value. Here, absorbance maxima corresponding to solely silicon-containing bonds are evidenced at ~1060, ~800, and ~450 cm^−1^. It is interesting to note the significant decrease in the intensity of ~950 cm^−1^ maxima, suggesting both its dual source in the non-calcined SiO_2_/PVA_0h aging sample (vibrations of bridged Si–OH and non-bridged free Si–O– due to higher hydrolysis) and the formation of pure SiO_2_ in the calcined SiO_2__0h aging sample (the sole presence of Si–OH bonds due to increased condensation occurred after calcination) [71,72,73]. These results confirm the formation of pure, inorganic silica-based networks.

With increasing the aging time to 1.5 h, no significant modifications are evident in the band corresponding to free hydroxyls (3000–3400 cm^−1^), or in the IR maxima corresponding to PVA-originating, carbon-containing bonds (Figure 2, purple). The slight increase in the overlapped vibrations of Si–O–C and Si–O–Si (~800 cm^−1^) may result from the formation of more linkages between TEOS and PVA in the solution during aging, thus indicating the increased production of SiO_2_/PVA composite. This latter observation is also supported by the slight shift of the Si–O–Si asymmetric stretching, indicating the intrinsic network rearrangement due to more polycondensation reactions [66,74].

The calcination of the SiO_2_/PVA_1.5h aging sample determines the mitigation of all the peaks matching PVA molecules, with the recorded infrared spectrum indicating the sole presence of intense silicon-containing bonds (Figure 2, orange). The blue-shift in the vibrations of Si−O−Si (both when compared to SiO_2_/PVA_1.5h and SiO_2__0h specimens) suggests the additional rearrangement of the inorganic network, and the formation of the pure and more compact silica-based sample [75,76].

**Figure 3:**
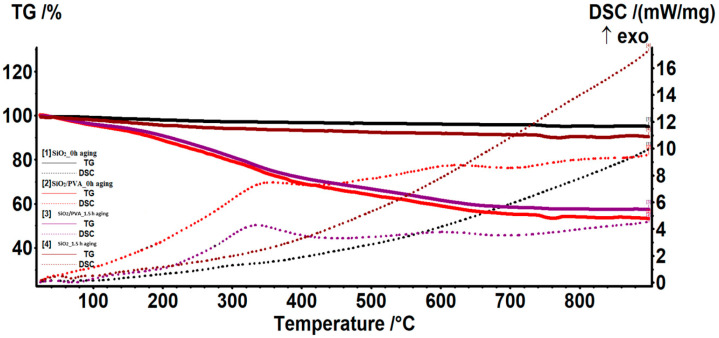
Thermal analysis for SiO_2_/PVA_0h aging (**red**), SiO_2__0h aging (**black**), SiO_2_/PVA_1.5h aging (**mauve**) and SiO_2__1.5h aging (**brown**).

To evaluate the thermal behavior of electrospun samples, complementary thermogravimetric analysis and differential scanning calorimetry investigations were performed and compared (Figure 3). Similar thermal patterns were recorded for non-calcined (SiO_2_/PVA_0h aging and SiO_2_/PVA_1.5h aging) and calcined (SiO_2__0h aging and SiO_2__1.5h aging) samples, respectively.

In the case of non-calcined samples (Figure 3, red and mauve), the presence of two exothermic effects can be observed between 250 and 900 °C, with maxima at 362.9/335.7 °C and 620.8/604.3 °C, attributed to non-aged and 1.5h-aged samples, respectively. These events, accompanied by mass losses of 31.73/26.06% and 15.9/11.96%, respectively, may be attributed to the oxidative processes of organic molecules and TEOS residuals, the loss of water from silanols, and the formation of SiO_2_ networks [77,78]. An additional mass loss of 4.67% is observed below 120 °C in the case of a 1.5 h-aged sample, due to the endothermic effect with a minimum at 70 °C caused by the evaporation of moisture water and residual volatile molecules [79,80]. Based on the thermal results, total mass losses of ~48% and ~43% are estimated for SiO_2_/PVA_0h aging and SiO_2_/PVA_1.5h aging samples, respectively.

**Figure 4:**
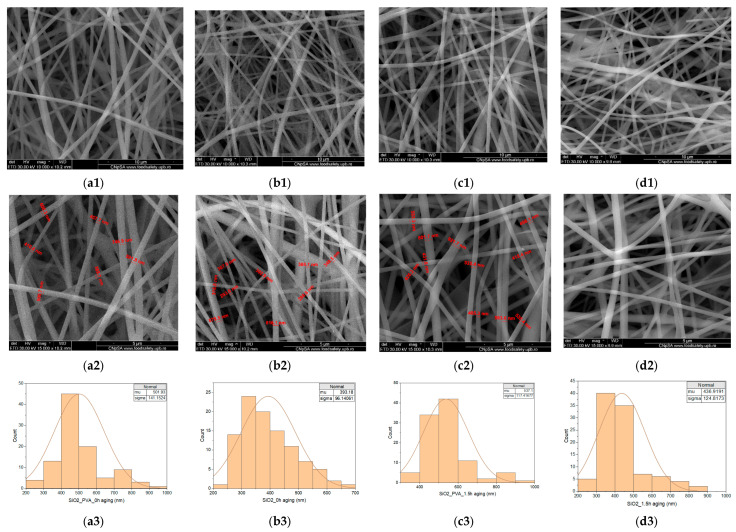
SEM images (**a1**–**d1**,**a2**–**d2**) and diameter size distribution (including average diameter values, (**a3**–**d3**)) recorded for SiO_2_/PVA_0h aging (**a1**–**a3**), SiO_2__0h aging, 500 °C (**b1**–**b3**), SiO_2_/PVA_1.5h aging (**c1**–**c3**) and SiO_2__1.5h aging (**d1**–**d3**).

For what concern the thermally treated specimens, a monotonous and slight total mass loss of ~4% and ~8% is evidenced for SiO_2__0h aging (Figure 3, black) and SiO_2__1.5h aging (Figure 3, brown), respectively. The evaporation of physioadsorbed water occurs right above 70 °C, followed by the continuous exothermic events assigned to the gradual condensation of surface silanols, which becomes more intense after 350 °C and is responsible for the densification of silica networks [81,82]. In accordance with the infrared data, these results indicate the predominant inorganic nature of calcined electrospun specimens. To investigate the morphological and dimensional characteristics of silica/PVA (Figure 4(a1,a2,c1,c2)) and silica (Figure 4(b1,b2,d1–d3)) samples, relevant SEM micrographs have been collected. As a general remark, the formation of macroporous fibrous networks consisting of uniformly distributed and randomly oriented, defect-free fibers with nano-size diameters is evident for all electrospun mats.

The formation of uniform nanofibers is noticed in the case of non-aged specimens, with a mean diameter of ~500 nm for SiO_2_/PVA_0h aging (Figure 4(a3)) and ~390 nm for SiO_2__0h aging (Figure 4(b3)). Besides smaller diameters, the calcination process also results in the formation of a denser and more compact mat, which confirms the intrinsic network rearrangement mentioned during IR results.

The formation of well-defined nanofibers is also noted with increasing aging time. In a similar way to the non-aged samples, the morphological and dimensional characteristics of the aged silica/PVA fibers are maintained (Figure 4(c3)), while uniformly organized nanofibers, with reduced dimensional distribution of their diameters, and denser appearance, results after calcination(Figure 4(d3)).

Additional information on the composition of electrospun networks was obtained using the energy-dispersive X-ray spectroscopy (EDS) accessory of the microscope. Besides oxygen, the collected spectra indicated the presence of very intense Si–Kα1 (~1.75 keV) and less intense Si–Kβ1 (~2 keV) peaks, in all samples. In the case of non-calcined samples, PVA-originating carbon was also identified. Complementary with the FT-IR spectra (evidencing only the presence of Si-containing bonds) and thermal analysis (indicating the predominant inorganic nature of calcined materials, with much reduced mass losses), these results confirm the formation of pure silica networks for SiO_2__0h aging and SiO_2__1.5h aging fibrous materials.

**Figure 5:**
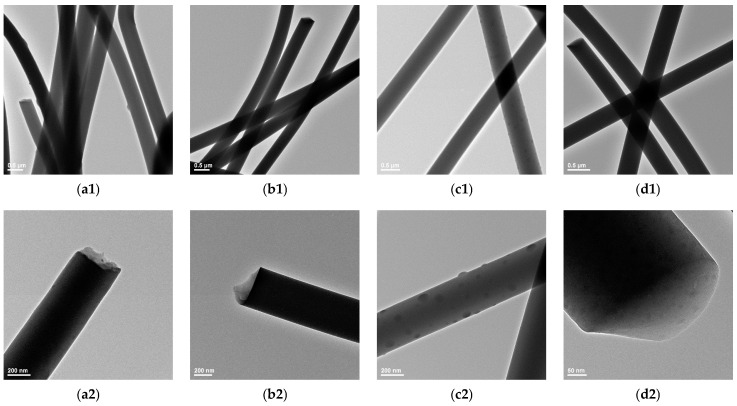
TEM images recorded for SiO_2_/PVA_0h aging (**a1**,**a2**), SiO_2__0h aging (**b1**,**b2**), SiO_2_/PVA_1.5h aging (**c1**,**c2**) and SiO_2__1.5h aging (**d1**,**d2**).

The intimate microstructure of prepared fibers was observed from the TEM images. The formation of nanofibers with a predominant smooth surface is observed for the non-aged and non-calcined sample (Figure 5(a1,a2)), with the presence of few PVA particulate structures. By contrast, nanofibers with exclusive smooth surfaces are observed in the case of SiO_2__0h aging material (Figure 5(b1,b2)), validating previous compositional results with respect to the formation of pure silica networks. More, TEM micrographs confirm SEM observations regarding the narrower diameter distribution of calcined nanofibers.

In the case of the 1.5h-aged and non-calcined specimen (Figure 5(c1,c2)), TEM images reveal the presence of fibers with highly particulate surfaces, suggesting their increased roughness. This is due to the abundant formation and entrapment of PVA particles during the synthesis process. This hypothesis is further supported by the absence of such particulate structures after calcination and the presence of nanofibers with smooth surfaces (Figure 5(d1,d2)). Additionally, TEM data validate the above-discussed results regarding the attainment of thermally stable and uniform networks based on pure silica.

### 3.2. Biological Evaluation of Nanofibrous Scaffolds

In light of modern medicine and considering the complex pathophysiology of natural bone, biologically inspired artificial constructs are optimal candidates for reviving the structural integrity and functionality of damaged bone tissues. Given the intricate composition, complex architecture, and biomechanical requirements of natural bones, inorganic nanomaterials—such as bioceramics, bioglasses, and oxides—are suitable choices for engineering advanced and successful bone substitutes [83,84].

**Figure 6:**
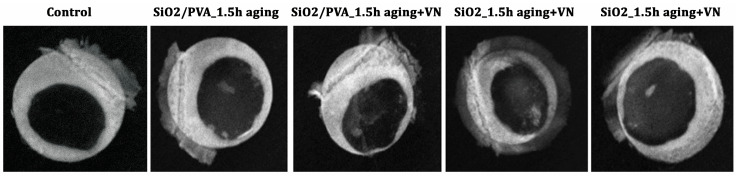
In vivo radiographs of the bone samples after 4 weeks of implantation of silica-based scaffolds.

**Figure 7:**
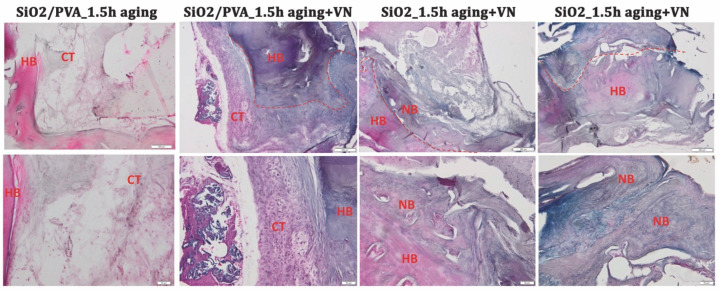
Histological aspect of the bone samples at 4 weeks post-surgery (H&E stain). Symbols: dotted line—separates healthy tissue from the defect; HB—host bone; CT—connective tissue; NB—new bone. Magnification ×4 and ×50.

**Figure 8:**
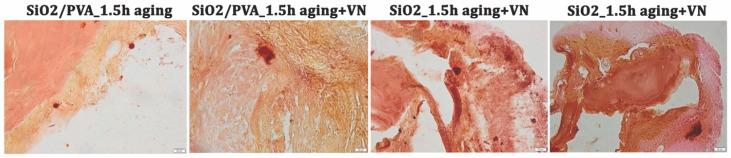
Histological aspect of the bone samples at 4 weeks post-surgery (Alizarin-Red stain). Magnification ×20.

Herein, the reparative and regenerative ability of the ES-generated mats has been evaluated at 4 weeks after the implantation of silica-based scaffolds in murine calvarial defects, using complementary X-ray imaging (Figure 6) and histological examination (Figure 7 and Figure 8).

As the radiological analysis shows (Figure 6), bone mineralization starts in week 4, extending from the edges of the defect to the center. Nevertheless, the radio-opacity in the mid-region of the defect is low, indicating less mineralization. A gradual increase in the new bone formation is observed for calcined silica fibers + VN, compared to other fibrous scaffolds and the control (bone defect without implant) (Figure 6).

At week 4 post-surgery, defect sites are filled with connective tissue, containing a scarce number of inflammatory cells, fibroblasts, and few blood vessels. Fibrous connective tissue is observed surrounding the silica-based scaffolds, which undergo a substantial change in the post-graft period.

Hematoxylin and Eosin (H&E) staining (Figure 7) indicates the cell population and connective matrix production, which starts from the periphery to the center. The new bone starts to be formed, especially in the case of calcined fibers, in the absence of PVA and mainly in the presence of vitronectin. All scaffolds were surrounded by a layer of fibrous tissue with collagen fibers and connective cells (mainly fibroblasts).

Alizarin-Red staining was used to detect calcium deposits and to confirm the osteogenic capacity of the silica-based electrospun scaffolds (Figure 8). Small-scale mineralization points appear at the periphery of bone defects and their size and ratio increase as follows: silica fibers < silica fibers + VN < calcined silica fibers < calcined silica fibers + VN.

The last group, corresponding to calvarial defects treated with SiO_2__1.5h aging nanofibrous scaffoldand vitronectin, has some extended mineralization points compared to all. This outcome is consistent with previous radiological results and is the synergistic result between the sole silica-based network (as this material is the second in line in terms of efficient mineralization and new bone formation) and vitronectin (multifunctional glycoprotein involved in bone cell attachment and migration, and subsequent bone tissue remodeling) [85,86].

**Figure 9:**
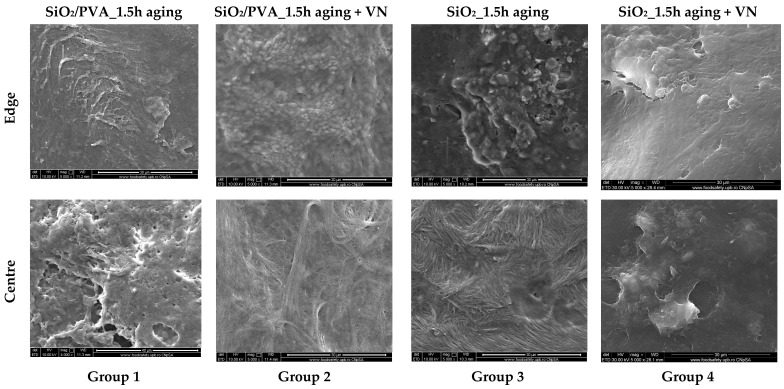
SEM images of the bone samples at 4 weeks post-surgery.

The SEM images of bone samples at 4 weeks post-surgery (Figure 9) show that osteoblast populations occur from edge to center, for all 4 tested groups. Samples on the surface of which more abundant cells are observed are those from groups that contain vitronectin (VN), due to the beneficial role of VN in promoting cell adhesion and differentiation. At the same time, the only sample at which the fibrous matrix can no longer be identified is part of group SiO_2__1.5h aging + VN.

Besides providing ECM-like compositional and microstructural characteristics, 3D electrospun scaffolds offer optimal and active support for cellular adhesion and proliferation, intercellular communication, and tuned cell physiology, finally resulting in performance-enhanced tissue substitutes. To closely resemble the complex architecture of natural bones and to properly match their requirements, ES-generated polymeric scaffolds reinforced with inorganic nanomaterials have been reported as promising solutions for bone repair and regeneration [87,88]. Adding inorganic nanofillers—such as bioceramics, bioglasses, and oxides—is a convenient strategy to modulate the events (biomineralization, osteoconductivity, and osteoinductivity) that are mandatory for bone TE [89,90,91].

Given the important regulatory role of silicon in skeletal physiology, silica-based nanomaterials have attracted tremendous attention for fabricating osteostimulative formulations and devices for bone restoration and replacement. Such constructed biomaterials generate strong interfaces with natural bone tissues and stimulate osteogenic events [92,93]. Due to the modulatory effects of SiO_2_ in bone homeostasis and its impressive drug carrier ability, electrospun polymeric scaffolds reinforced with nano-silica have been successfully evaluated for bone reconstruction [94,95].

Herein, composite SiO_2_/PVA and sole SiO_2_ nanofibrous scaffolds have been developed by electrospinning, and their bone regeneration ability has been demonstrated on murine calvarial defects. In terms of mineralization and new bone formation, superior outcomes have been evidenced for the calcined fibrous samples, with better results in the case of 1.5 h-aged specimen. Still, the osteogenic events have been significantly boosted after the combined treatment with vitronectin (VN). Our results, which confirm the potential use of high-purity electrospun silica nanofibers in bone tissue engineering applications, are in compliance with previous studies on nano-silica fibers. More than just improving the mechanical properties of polymeric scaffolds, nanofibrous SiO_2_ promotes the osteogenic differentiation of stem cells and stimulates angiogenic events, resulting in promising composite constructs for bone regeneration [96,97,98,99].

## 4. Conclusions

The synthesis and evaluation of silica (SiO_2_) nanofibrous scaffolds using tetraethyl orthosilicate (TEOS) and polyvinyl alcohol (PVA) during electrospinning and subsequent calcination are herein reported. The compositional and microstructural features of the obtained mat were complementary and evaluated using infrared and thermal studies and advanced microscopic techniques. The formation of pure silica networks, consisting of uniformly distributed and randomly oriented nanofibers with smooth surfaces was observed after calcination at 500 °C, for 2h. Then, the samples were evaluated for their potential use in bone tissue engineering using a murine animal model. Superior results, in terms of mineralization and new bone formation, were obtained for calcined silica fibrous scaffolds (SiO_2__0h aging and SiO_2__1.5h aging). Moreover, vitronectin-reinforced calcined networks displayed accelerated mineralization and promoted new bone formation by increasing cell adhesion to the fibers. Our results validated the use of electrospun silica nanofibers as efficient scaffolding candidates for bone tissue engineering applications.

## Data Availability

Data are available at the authors.
